# Inflammatory profiling for precision neurorehabilitation after traumatic brain injury and stroke: a narrative review

**DOI:** 10.3389/fneur.2026.1854973

**Published:** 2026-09-24

**Authors:** Rocco Salvatore Calabrò, Andrea Calderone, Alessio Ardizzone, Irene Ciancarelli, Giovanni Morone, Simona Cammaroto, Carmela Rifici, Angelo Quartarone

**Affiliations:** 1 Department of Neurorehabilitation, IRCCS Centro Neurolesi Bonino Pulejo, Messina, Italy; 2 Departmental Faculty of Medicine, UniCamillus-Saint Camillus International University of Health Sciences, Rome, Italy; 3 Department of Life, Health and Environmental Sciences, University of L'Aquila, L'Aquila, Italy; 4 LIFE Institute for Research and Health Care Santa Lucia IRCCS, Rome, Italy

**Keywords:** biomarkers, neuroinflammation, neuroplasticity, neurorehabilitation, precision rehabilitation, rehabilitation responsiveness, stroke, traumatic brain injury

## Abstract

**Introduction:**

Recovery after traumatic brain injury (TBI) and stroke is heterogeneous, even among patients with similar lesion burden, clinical severity, and rehabilitation exposure. Neuroinflammation is a biologically plausible contributor, but evidence that inflammatory state modifies the effect of a specific rehabilitation treatment remains limited.

**Methods:**

This structured narrative review, informed by the Scale for the Assessment of Narrative Review Articles (SANRA), evaluates inflammatory biomarkers as candidate modifiers of response to therapeutic exercise, robotics, virtual reality (VR), telerehabilitation, and non-invasive brain stimulation (NIBS). PubMed, Scopus, Embase, and Web of Science were searched from inception through March 23, 2026. Eligibility, selection logic, evidence charting, and a population, intervention, comparator, and outcome framework are reported in tabular Supplementary Material. Evidence was classified as direct interventional, observational human, preclinical or mechanistic, or conceptual inference.

**Results:**

Inflammatory state is not a simple harmful vs. beneficial binary: timing, persistence, cellular context, structural reserve, systemic physiology, and treatment dose shape interpretation. Blood and cerebrospinal fluid markers, including interleukin-6, C-reactive protein, glial fibrillary acidic protein, neurofilament light chain, and composite immune indices, can support clinically meaningful, longitudinal biological phenotyping, but sampling and assay heterogeneity limit clinical translation. A small randomized stroke study linking very early exercise, serial inflammatory changes, and functional outcomes provides emerging direct evidence, although it did not establish biomarker-defined treatment effect modification. For stroke, human biomarker and rehabilitation evidence is comparatively developed, but prospective interaction testing remains scarce. For TBI, evidence is substantially weaker and predominantly indirect or mechanistic.

**Discussion:**

Accordingly, the proposed multimodal framework is a hypothesis for prospective trial enrichment, not a validated treatment matching algorithm. Its clinical value depends on standardized serial sampling, dose documentation, rehabilitation-specific outcomes, and externally validated prospective interaction testing within conditions. No single blood test or panel can currently identify rehabilitation responders, and inflammation-guided treatment assignment is not ready for routine care.

## 1 Introduction

Traumatic brain injury (TBI) and stroke remain leading causes of death, disability, and long-term dependence worldwide, and their combined burden is rising in parallel with population aging, survival after severe acute care, and the growing number of individuals living with chronic neurological deficits (1–3). The major clinical challenge is no longer confined to survival. It concerns how to move patients from biological stabilization to meaningful functional recovery. Rehabilitation therefore occupies a central position in both conditions, yet contemporary rehabilitation practice still relies heavily on clinical phenotype, impairment severity, therapist expertise, and pragmatic service constraints rather than on biologically grounded stratification (4–6).

This gap is particularly relevant because recovery is not uniform. Patients with apparently comparable lesions, baseline disability, and therapy exposure can show different motor, cognitive, language, and participation trajectories after TBI (3). Response heterogeneity affects conventional therapy, exercise, robotics, virtual reality (VR), telerehabilitation, and non-invasive brain stimulation (NIBS). Stroke trials show improvement in both treatment and comparison groups, suggesting that average effects may be diluted when biologically distinct patients are analyzed as one population (5, 6). Mixed injury mechanisms, extracranial trauma, premorbid vulnerability, psychiatric burden, and chronic systemic dysfunction further complicate TBI prognosis and treatment selection (7, 8).

Neuroinflammation is a compelling candidate modifier of this heterogeneity. Following stroke and TBI, inflammatory signaling begins within minutes and evolves across recovery phases through innate and vascular mechanisms (7, 8). Microglia, astrocytes, endothelial cells, peripheral immune cells, and circulating mediators participate in this response (9, 10). Inflammation is not intrinsically maladaptive: appropriately regulated activity contributes to debris clearance, synaptic remodeling, vascular adaptation, and repair (11, 12). Excessive or persistent activity may instead amplify blood-brain barrier (BBB) disruption, white-matter injury, metabolic stress, maladaptive pruning, and chronic neurodegeneration (13–16). The core rehabilitation question is therefore not whether inflammation occurs, but whether a defined inflammatory state changes the benefit derived from training, stimulation, practice, or environmental enrichment.

This question has become more pressing as rehabilitation technologies have advanced. Robotic platforms can increase repetition and dose; VR can enhance motivation, immersion, and task salience; telerehabilitation can extend care into the home; and NIBS can alter cortical excitability and network dynamics. These interventions act on neural systems whose responsiveness may be constrained or facilitated by biological context. If inflammatory biology influences plasticity, it could also influence rehabilitation response, but this remains a hypothesis. Persistent inflammation, astroglial reactivity, systemic catabolism, or adaptive inflammatory resolution may interact with treatment timing and structural reserve (11, 13, 17, 18); none currently defines a validated treatment-selection rule.

The translational literature has begun to move in this direction, but progress remains incomplete. Several recent reviews have summarized neuroinflammation after stroke or TBI, and others have cataloged blood biomarkers or technology-assisted rehabilitation in relative isolation. What remains less developed is a synthesis specifically organized around rehabilitation responsiveness. That distinction is critical. A biomarker associated with acute severity, recurrent vascular risk, or broad outcome is not automatically a biomarker of treatment-effect modification. Direct links between inflammatory signatures and response to robotics, VR, exercise, telerehabilitation, or neuromodulation remain relatively uncommon, especially in TBI. The field therefore has an expanding biological foundation, but a more limited evidence base for treatment matching.

The present review addresses that gap by examining neuroinflammation as a potential modifier of rehabilitation responsiveness after TBI and stroke. This is not a generic overview of post-injury inflammation. Its specific aim is to connect inflammatory biology with responder heterogeneity, rehabilitation timing, modality selection, and enrichment logic for future trials. We focus on the temporal biology of inflammation, the interface between inflammation and neuroplasticity, the strengths and weaknesses of available biomarker evidence, and the implications for major rehabilitation modalities. We then propose a translational framework that integrates inflammatory markers with imaging, neurophysiology, clinical phenotype, and digital outcomes to guide trial enrichment and individualized care. Figure 1 synthesizes this temporal logic by aligning the hyperacute, acute, subacute, and chronic phases of TBI and stroke with their principal biological events, inflammatory and glial dynamics, and evolving rehabilitation opportunity windows. By placing early symptom or medical instability alongside later training tolerance and long-term adaptation, the figure clarifies why rehabilitation timing should be interpreted as a biological rather than purely logistical decision.

**Figure 1:**
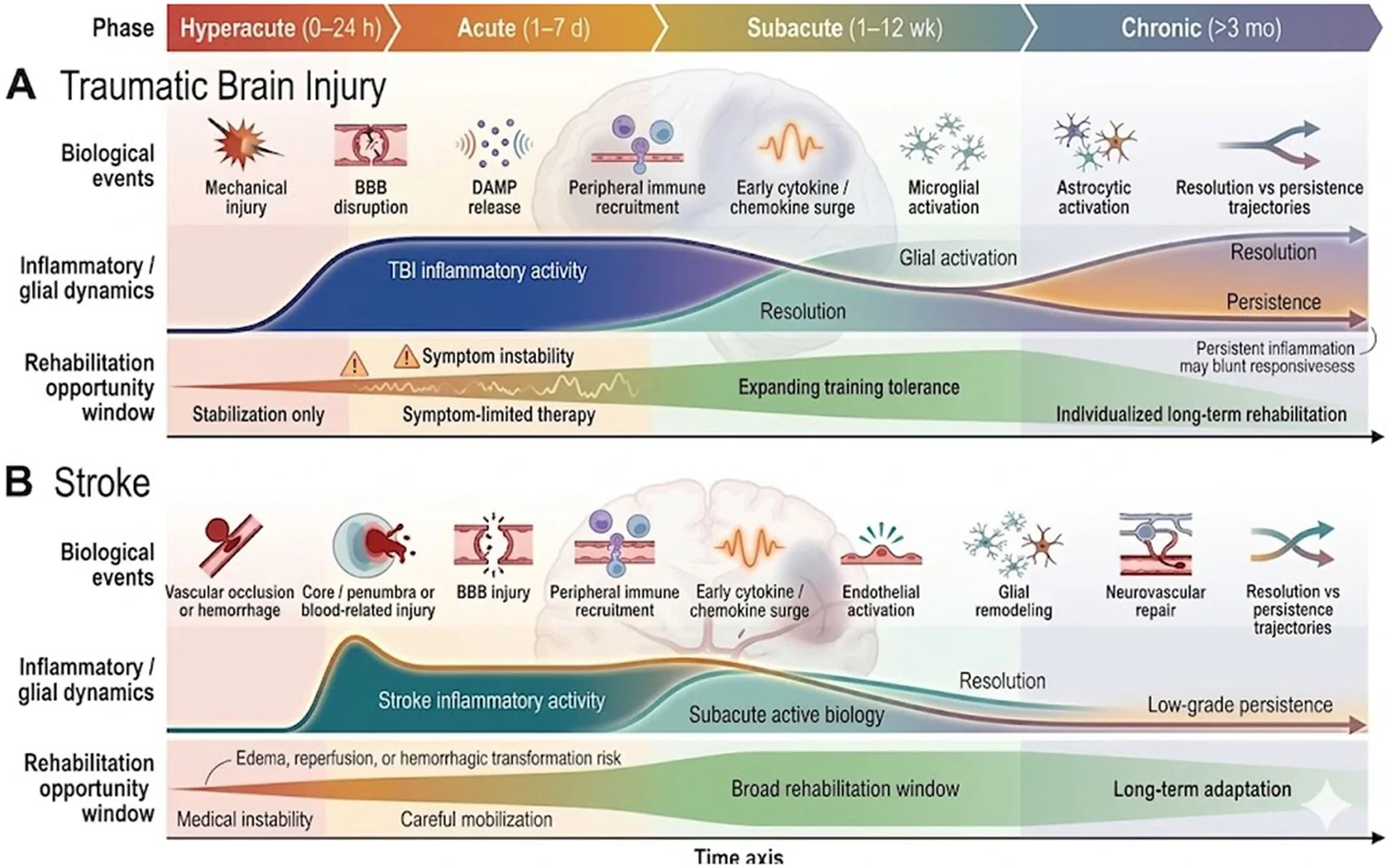
Temporal inflammatory and glial dynamics and rehabilitation opportunity windows after traumatic brain injury and stroke. **(A)** Traumatic brain injury and **(B)** stroke are aligned across hyperacute, acute, subacute, and chronic phases. The schematic relates principal biological events and resolution-vs.-persistence trajectories to evolving rehabilitation opportunities; it does not define validated biomarker thresholds or treatment-selection rules. BBB, blood-brain barrier; DAMP, damage-associated molecular pattern; TBI, traumatic brain injury.

## 2 Methods

### 2.1 Review design and question framework

This work was designed and reported as a structured narrative review because the objective was to integrate biological, biomarker, and rehabilitation evidence rather than estimate a pooled intervention effect. The review was informed by the Scale for the Assessment of Narrative Review Articles (SANRA), used as a quality framework rather than as a substitute for systematic-review methodology. The question was organized using a population, intervention or exposure, comparator, and outcome (PICO) framework adapted to an etiologically broad narrative synthesis. The population included adults with stroke or TBI; exposures included inflammatory profiles and rehabilitation modalities; comparators included alternative biological states, doses, modalities, or usual care when available; and outcomes emphasized treatment gain, tolerance, retention, and real-world transfer. The operational PICO scope is reported in Supplementary Table S1.

### 2.2 Information sources and search architecture

PubMed, Scopus, Embase, and Web of Science were searched from database inception through March 23, 2026; source functions and coverage are summarized in Supplementary Table S2. A single Boolean architecture combined three concept blocks: condition terms for stroke or TBI; inflammatory, glial, neuroaxonal, vascular, and systemic immune-marker terms; and recovery, neuroplasticity, rehabilitation-response, or modality terms. Targeted terms covered exercise, robotics, virtual reality, telerehabilitation, non-invasive brain stimulation, imaging, and digital monitoring. Reference lists of high-relevance reviews and clinical or translational studies were checked to identify additional sources. The database, platform, final search date, and verbatim-search fields are organized in Supplementary Table S3.

### 2.3 Eligibility and evidence selection

Sources were eligible when they informed at least one linked question: the temporal evolution of inflammation after stroke or TBI; interactions between inflammation and plasticity or repair; biomarker-supported rehabilitation phenotyping; or differential response to therapeutic exercise, robotics, virtual reality, telerehabilitation, or non-invasive brain stimulation. Human interventional and observational studies, systematic or scoping reviews, meta-analyses, consensus or methodological reports, and selected preclinical studies with a direct mechanistic link were considered. Sources focused solely on diagnosis, acute treatment, or mortality were retained only when needed to define biological phase, measurement validity, or prognostic limits. Reports without a clear connection to inflammation, recovery biology, rehabilitation exposure, or response measurement were excluded from the synthesis. Recent evidence was prioritized, while older studies were retained only when foundational or not superseded. Eligibility, prioritization, and exclusion rules are specified in Supplementary Table S4.

### 2.4 Data charting and evidence-level classification

For each source, the synthesis charted condition and subtype, recovery phase, study design, sample context, biomarker or biological domain, rehabilitation modality and documented exposure when available, outcome domain, and whether the evidence addressed prognosis, treatment response, or treatment-effect modification. Claims were then classified as direct interventional evidence, observational human evidence, preclinical or mechanistic evidence, or conceptual inference. Direct interventional evidence required a prospective rehabilitation study relating biomarker state or change to a defined intervention and outcome; a biomarker-by-treatment interaction was considered stronger than parallel biomarker and outcome change. This classification, including permitted interpretations and principal limitations, is reported in Supplementary Table S5.

### 2.5 Narrative synthesis, auditability, and limitations

Synthesis was purposive, iterative, and condition specific. Evidence supporting efficacy of a rehabilitation modality was separated from evidence that inflammation modifies its effect, and prognostic association was not interpreted as treatment selection. Citation clusters were limited by assigning references to the individual claims they supported. Because this was a narrative review, Preferred Reporting Items for Systematic Reviews and Meta-Analyses (PRISMA) screening counts, protocol registration, duplicate independent screening, formal risk-of-bias scoring, and meta-analysis were not undertaken; no record count was reconstructed retrospectively. These design boundaries restrict exhaustiveness and causal inference but preserve an auditable distinction between evidence and hypothesis. SANRA alignment and the corresponding manuscript locations are mapped in Supplementary Table S6.

### 2.6 Artificial intelligence assistance

Artificial intelligence assistance was limited to language refinement, structural editing, reference-formatting checks, and assistance with conceptual figure rendering using OpenAI Codex within ChatGPT and OpenAI image-generation functionality (5.6 version; OpenAI, San Francisco, CA, USA; accessed March 2026). All database queries, evidence selections, scientific interpretations, references, and visual content were critically reviewed and approved by the authors, who take full responsibility for the final manuscript.

## 3 Biological logic of inflammation-responsive recovery

### 3.1 Temporal architecture of post-injury inflammation

Inflammation after stroke and TBI begins before the clinician can reasonably intervene and can persist after the acute lesion has stabilized on conventional imaging. Damage-associated molecular patterns and cellular debris trigger innate immune signaling after TBI (7, 8), while vascular disruption and neurovascular-unit stress are especially prominent after stroke (9, 10). Microglia are among the earliest responders (11, 19), but their activation is not binary and the classically activated vs. alternatively activated framework is insufficient. Time-dependent phenotypes may support phagocytosis and repair or sustain cytokine release, oxidative injury, and maladaptive circuit remodeling; chronic inflammatory and metabolic processes can extend this biological response (20, 21).

Astrocytes are equally important but are often discussed too generically. In the acute phase they support ion buffering, metabolic stabilization, glutamate handling, and barrier regulation (9, 10). Reactive astroglial states can also amplify tissue injury, alter extracellular matrix composition, and contribute to chronic white-matter inflammation or scar-related constraints on axonal remodeling (14, 21, 22). Therapies that rely on experience-dependent plasticity assume that damaged networks retain capacity for functional reorganization. Persistent glial dysregulation may narrow that window by altering synaptic homeostasis, trophic support, and communication among neurons, glia, and endothelial cells.

The vascular interface is another major determinant of phase-specific inflammatory biology. Stroke by definition emerges from vascular failure, while TBI frequently produces microvascular damage, endothelial activation, and BBB breakdown as part of secondary injury. In both conditions, barrier dysfunction enables peripheral immune recruitment and exposes brain tissue to circulating inflammatory mediators whose concentration and timing may differ substantially across patients (10, 14, 15). This is one reason why apparently similar lesions can have biologically different downstream trajectories. The systemic response to injury matters. Peripheral monocytes, neutrophils, lymphocyte dynamics, stress hormones, infection burden, and extracranial trauma can all modify the inflammatory phenotype that the injured brain experiences.

Stroke and TBI differ in ways that matter for rehabilitation stratification. TBI is especially heterogeneous across injury mechanisms and systemic contexts (3, 7). In ischemic stroke, inflammatory biology is shaped by reperfusion status, infarct volume, hemorrhagic transformation, vascular territory, and vascular risk (9); hemorrhagic stroke adds blood-degradation products, perihematomal edema, and neurovascular irritation. Moderate-to-severe TBI can combine focal and diffuse injury (14, 16), while extracranial stress and chronic inflammation add further heterogeneity (17, 19). Neurobehavioral and neurodegenerative sequelae broaden the chronic phenotype (20–22). These associations do not establish rehabilitation treatment-effect modification.

The temporal structure of inflammation is particularly relevant. Acute inflammatory activity is often treated as uniformly harmful, yet early phagocytic and repair-associated signaling is necessary for tissue clearance and stabilization. The translational problem emerges when inflammatory responses are excessive, poorly resolved, or spatially mismatched to evolving repair needs. A patient exposed to very early high-dose rehabilitation during a period of marked metabolic instability and unresolved neuroinflammation may not respond as predicted by baseline impairment alone. By contrast, a patient whose inflammatory profile is transitioning toward resolution may be entering a biologically permissive period for intensive motor training, enriched practice, or stimulation-based interventions. This temporal logic is not yet operationalized in routine neurorehabilitation, but it is central to the responder/non-responder framework proposed in this review.

The cellular architecture of inflammation is intertwined with non-neurological physiology. TBI can provoke endocrine dysfunction and systemic catabolism (3, 7), while sleep disruption, autonomic imbalance, and chronic pain may feed back into inflammatory signaling and reduce readiness for rehabilitation (20–22). Stroke survivors may similarly carry inflammatory amplification from obesity, diabetes, atrial disease, infection, frailty, or post-acute medical complications. The inflammatory state relevant to rehabilitation is therefore an organism-level condition with neural, vascular, endocrine, and behavioral components. A responder model that ignores this context risks collapsing meaningful heterogeneity into noise.

From a clinical perspective, the major implication is straightforward. Post-injury inflammation should not be conceptualized as a background process occurring in parallel to rehabilitation. It is part of the biological substrate on which rehabilitation acts. When this substrate is highly unstable, chronically dysregulated, or poorly resolved, the same therapeutic dose may yield less gain, greater fatigue, more fluctuations, or different risk-benefit trade-offs than in a patient with a more favorable inflammatory profile. This does not imply that rehabilitation should be withheld when inflammation is present. Rather, it suggests that the timing, intensity, and mode of rehabilitation may need to be matched to inflammatory phase rather than assumed to be biologically neutral.

### 3.2 Inflammatory control of plasticity-relevant repair circuits

The link between neuroinflammation and rehabilitation responsiveness becomes more persuasive when viewed through neuroplasticity. Functional recovery depends on synaptic scaling, dendritic remodeling, and axonal sprouting (11, 23, 24), as well as remyelination, network recruitment, and compensatory reweighting of surviving pathways (25–27). None occurs in an immunologically silent environment. Microglia participate in synaptic pruning and structural remodeling; astrocytes regulate transmission and metabolic support; and cytokines influence excitability, trophic signaling, and plasticity-related gene expression. The same mediator that supports adaptive remodeling at one concentration or timepoint may impair it at another.

This duality is clear in experimental stroke literature. Microglial activation contributes to early ischemic injury and to later repair or synaptic remodeling (11). Immunological pathways can support poststroke recovery (23), whereas chronic microglial metabolic reprogramming may hinder neurorestoration (24). TBI exercise immunology provides a parallel conceptual bridge (28), and clinical stroke syntheses connect training with functional outcomes (29–31). Preclinical TBI work implicates exercise-related brain-derived neurotrophic factor signaling (32), while stroke studies link aerobic or intensive training with neuroprotection, inflammatory modulation, and repair (33–35). These studies support biological plausibility; they do not by themselves demonstrate a human biomarker-by-rehabilitation interaction.

Exercise literature after brain injury provides similar clues. Mechanistic work links exercise with trophic signaling and plasticity after brain injury (28, 30), while experimental and early clinical studies address recovery-related effects (32, 33). Recent syntheses evaluate human neuroplasticity markers and timing-dependent exercise effects after stroke (34, 35). This matters because rehabilitation dose is not synonymous with gain. High-volume treatment in a biologically permissive period may differ from the same nominal dose during unresolved illness, low arousal, endocrine dysfunction, or metabolic stress. Whether measured inflammation explains this state dependence remains to be tested prospectively.

White-matter integrity further complicates this picture. Rehabilitation responsiveness often depends on residual corticospinal and network reserve, but white matter is vulnerable to inflammatory damage after stroke and TBI (10, 21, 22). Persistent inflammation may impair oligodendroglial function, myelin integrity, and long-range connectivity (25, 26), thereby reducing the ability to translate training into stable behavioral improvement. This does not mean that severe inflammatory injury precludes recovery; it means that the biological cost of recovery may be higher, the required pathway may differ, and plateaus may emerge for reasons not visible on routine bedside examination.

Another important but under-discussed issue is the relationship between inflammation and compensation. Patients may improve on clinical scales through compensatory strategies rather than through restitution of the affected neural system. Inflammatory states could plausibly bias the balance between these recovery modes. If a persistent inflammatory burden limits restoration of damaged corticospinal circuits, patients may rely more heavily on compensatory trunk movements, altered task selection, or contralesional recruitment. Such adaptation can be functionally useful, but it may not represent the same biological response to therapy as true restitution. This distinction matters when interpreting trials. Two patients may show similar short-term gains on a functional measure while reaching that endpoint through biologically distinct pathways, one more consistent with adaptive circuit restoration and the other with compensation under constraint.

These considerations support studying inflammation as a candidate modifier of responsiveness rather than only as a prognostic marker. A prognostic marker predicts outcome regardless of treatment; a modifier changes the effect of a treatment. The current literature does not establish that any inflammatory biomarker modifies response to a specific rehabilitation modality in routine care. Cellular biology, exercise research, mechanistic stroke studies, and chronic TBI work provide a rationale for prospective interaction testing, but not for treatment assignment. Figure 2 therefore presents a hypothesis-generating mechanistic model in which inflammatory and glial signals may interact with repair circuits and structured rehabilitation inputs. It should not be read as a validated causal pathway, responder classifier, or dosing rule.

**Figure 2:**
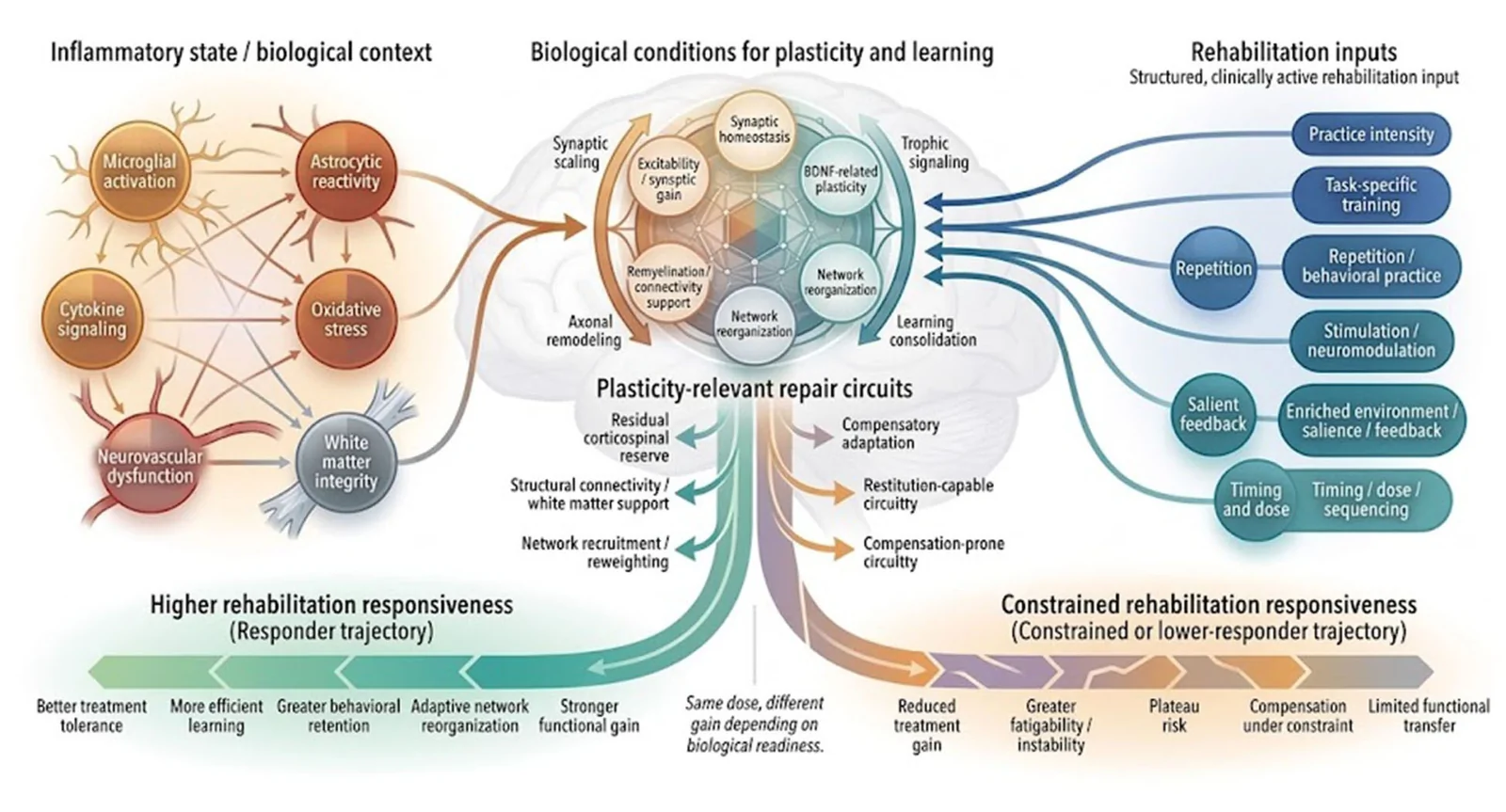
Mechanistic model linking neuroinflammation, neuroplasticity, and rehabilitation responsiveness after traumatic brain injury and stroke. Inflammatory and vascular conditions may influence plasticity-relevant repair circuits, while rehabilitation dose, task specificity, repetition, feedback, stimulation, and timing provide the training inputs. Divergent responder and constrained-response trajectories are conceptual hypotheses rather than established causal pathways. BDNF, brain-derived neurotrophic factor; TBI, traumatic brain injury.

## 4 Biomarker profiling for rehabilitation stratification

### 4.1 From soluble mediators to composite inflammatory panels

The biomarker literature relevant to rehabilitation stratification is broad, but its translational value depends on moving beyond simple cataloging. A clinically useful marker must do more than correlate with injury severity. It must clarify biological phase, identify a subgroup with a distinct recovery trajectory, improve patient selection, or help explain why a given intervention works for some patients and not for others. From this perspective, the most informative biomarkers are not necessarily the most disease-specific. They are the ones that connect inflammatory biology to clinically meaningful recovery processes.

In stroke, contemporary reviews define the acute blood-biomarker landscape (36), while human inflammatory evidence and recent prognostic syntheses refine its interpretation (37, 38). Diagnostic differentiation has been examined separately (39, 40). Candidate inflammatory domains include cytokines and acute-phase proteins (41–43), stress-related signals (44, 45), and composite systemic indices (46–48). Endothelial, chemokine, and neurovascular markers provide additional biological context (49, 50). This literature is predominantly diagnostic or prognostic: it does not by itself show that a marker changes the comparative benefit of a rehabilitation treatment.

The TBI literature is more heterogeneous. Systematic reviews describe inflammatory signals after mild TBI (51, 52), whereas current National Institute for Neurological Disorders and Stroke recommendations emphasize standardized interpretation of blood-based biomarkers across injury contexts (53). Broader clinical syntheses address neuroinflammatory evidence (54) and diagnostic applications (55, 56). Neurochemical and chronic-fluid biomarkers add information about disease monitoring (57, 58). Extracellular vesicles and cerebrospinal-fluid markers extend the candidate landscape (59–62), while oxidative-stress evidence and a contemporary living meta-analysis provide additional context (63, 64). Interleukin 6 has prognostic support (65), and composite immune indices have been associated with outcome (66, 67). Nevertheless, rehabilitation exposure and pre-specified biomarker-by-treatment interaction are rarely measured; TBI-specific treatment-matching claims therefore remain largely indirect.

This limitation points to a crucial distinction. A biomarker can be prognostically valuable without being useful for treatment selection. Glial fibrillary acidic protein (GFAP) may reflect astroglial injury, neurofilament light chain (NfL) may reflect axonal damage, and C-reactive protein (CRP) may reflect systemic inflammatory burden. None alone can establish whether a patient is more likely to benefit from robotics than intensive conventional therapy, or from telerehabilitation rather than supervised clinic-based care. Direct interventional evidence for such matching remains limited. Multimarker panels may capture inflammatory, glial, neuroaxonal, vascular, and systemic physiology more plausibly than an isolated value, but their incremental utility must be tested. Candidate domains are summarized in Table 1.

**Table 1:** Biomarker domains, phase, condition, standardized evidence level, candidate rehabilitation relevance, and current strength of support.

Biomarker or domain	Most informative phase	Condition(s)	Evidence level for rehabilitation-response claim	Candidate rehabilitation relevance	Current support and representative references
Interleukin 6 (IL-6) and related cytokines	Acute to subacute; selected chronic persistence	Ischemic or hemorrhagic stroke; mild-to-severe traumatic brain injury (TBI)	Observational human evidence; very limited direct interventional evidence	Candidate marker of biological stress, dose tolerance, and phase readiness; treatment-effect modification remains untested	One small stroke exercise trial provides limited direct evidence (33). Prognostic support is otherwise moderate (41–43, 65); treatment matching remains unvalidated
High-sensitivity C-reactive protein (hs-CRP) and acute-phase proteins	Acute to chronic	Stroke more than TBI	Observational human evidence	Broad vascular/systemic context rather than a modality selector	Acute-phase evidence (42, 43) and systemic-index studies (46–48) support burden estimation; rehabilitation specificity remains low
Glial fibrillary acidic protein (GFAP)	Hyperacute to subacute	TBI; hemorrhagic stroke	Observational human evidence	Contextualizes astroglial injury and structural burden; no validated dose-selection role	Strong for injury characterization; low for modality-specific matching (56–58)
Neurofilament light chain (NfL)	Subacute to chronic	TBI and stroke	Observational human evidence	May contextualize axonal injury and realistic pacing; direct response prediction is untested	Moderate for neuroaxonal burden; low for treatment selection (57–59, 64)
Cerebrospinal fluid (CSF) inflammatory markers	Acute to subacute	Moderate-to-severe TBI; selected stroke cohorts	Observational human plus mechanistic evidence	Supports severe-injury phenotyping but has limited rehabilitation scalability	Moderate mechanistic value; limited clinical portability (57, 62)
Neutrophil-to-lymphocyte ratio (NLR), systemic immune-inflammation index (SII), systemic inflammatory response index (SIRI), and related blood-count indices	Acute to chronic; preferably serial	Stroke and TBI	Observational human evidence; prognostic, not treatment-modifying	Low-cost systemic-risk covariates; not stand-alone rehabilitation stratifiers	Stroke evidence (46–48) and TBI evidence (66, 67) are prognostic. Critical-stroke data (122) do not support treatment matching
Endothelial, chemokine, and neurovascular markers	Hyperacute to subacute	Stroke more than TBI	Preclinical/mechanistic plus indirect human evidence	May contextualize reperfusion, BBB injury, and vascular constraints on early mobilization	Emerging support; insufficient standardization (39, 40, 49, 50)
Cytokine or multi-analyte panels	Phase-dependent serial sampling	Stroke and TBI	Emerging observational/translational evidence	More plausible than single markers for research stratification	Promising but not clinically validated for treatment assignment (44, 45, 53, 68)
Integrated biomarker, imaging, and neurophysiology panels	Subacute to chronic	Stroke and TBI	Conceptual inference; limited direct testing	Candidate framework for trial enrichment and hypothesis testing	Multimodal rationale is supported conceptually (68, 112, 115). Rehabilomics and digital monitoring add context (118, 120, 121); no implementation readiness

The evidence-level column distinguishes observational, mechanistic, interventional, and conceptual support for rehabilitation-response claims. BBB, blood-brain barrier; CRP, C-reactive protein; CSF, cerebrospinal fluid; GFAP, glial fibrillary acidic protein; hs-CRP, high-sensitivity C-reactive protein; IL-6, interleukin-6; NfL, neurofilament light chain; NLR, neutrophil-to-lymphocyte ratio; SII, systemic immune-inflammation index; SIRI, systemic inflammatory response index; TBI, traumatic brain injury.

Panels are especially attractive because the responder/non-responder problem is multidimensional. A patient can fail to respond because the target network is too damaged, because the intervention dose is insufficient, because adherence is poor, because the outcome chosen is not sensitive to the right domain, or because the biological milieu is not permissive for plasticity. Biomarkers that are interpreted in isolation can only illuminate a fraction of this space. A biologically richer approach would combine inflammatory data with indicators of corticospinal reserve, lesion topology, cognition, fatigue, mood, sleep, and treatment exposure. In that model, inflammation is neither ignored nor overprivileged. It becomes one axis in a clinically interpretable multidomain signature.

An additional translational advantage of biomarker panels is that they could help define rehabilitation timing rather than only rehabilitation candidacy. A persistently elevated inflammatory profile may identify a subgroup in which early gains are likely to be slower, variability is likely to be higher, and the sequence of interventions may matter more than the absolute dose of any single approach. Conversely, a profile suggesting inflammatory resolution and preserved structural reserve might justify more aggressive motor practice or stimulation-based protocols. This type of reasoning remains prospective rather than proven, but it is precisely the level at which biomarker research becomes clinically useful.

### 4.2 Timing, assay heterogeneity, and phase-specific interpretation

The major obstacle to using inflammatory biomarkers for rehabilitation stratification is interpretive instability rather than a shortage of candidates. The same molecule can mean different things depending on timing, measurement platform, specimen compartment, comparator, and target outcome. Stroke biomarkers vary across acute clinical contexts (36, 37), mild-TBI signals vary with injury and sampling features (51, 52), and chronic or cerebrospinal-fluid markers introduce additional temporal and compartmental complexity (59, 62). This problem becomes sharper for rehabilitation response because gain depends on treatment timing, dose, and biological context more directly than broad disability or mortality outcomes.

Timing is the most obvious source of heterogeneity. In acute ischemic stroke, a marker may reflect occlusion, reperfusion, infarct growth, hemorrhagic transformation, or systemic stress; after hemorrhagic stroke it may instead track hematoma burden, edema, or blood-breakdown biology. After TBI, hyperacute values may be dominated by primary injury and extracranial trauma (51, 52) or by acute-care exposures (55, 58). Chronic values may instead reflect extracellular-vesicle or neurochemical signals (59, 60), biomarker compartment (62), oxidative stress and ongoing inflammation (63, 65), or systemic indices and rehabilitation context (66–68). A single threshold carried across phases is therefore unlikely to be meaningful.

Assay variability further complicates interpretation. Serum and plasma are not interchangeable, platforms yield different absolute concentrations, and preprocessing, storage, hemolysis, or freeze-thaw cycles can affect reliability. Cerebrospinal fluid may better reflect central processes but is invasive and less scalable. Stroke biomarker reviews document substantial measurement heterogeneity (36), including variability across stress-related and composite signals (44, 45). Comparable issues appear in mild-TBI syntheses (51, 52) and in current prognostic meta-analyses (64, 65). Nominally identical biomarkers may therefore represent non-equivalent biological constructs.

Clinical phenotyping is often equally inconsistent. Stroke studies variably mix ischemic and hemorrhagic cases, acute treatment exposures, recurrent events, and different subtype distributions. TBI studies vary in severity, mechanism, imaging burden, extracranial injury load, and chronicity. Many biomarker studies are designed around mortality or broad functional scales rather than around rehabilitation-specific outcomes such as dose tolerance, rate of gain, retention of training effect, or response to a defined therapeutic modality. This mismatch is central. A marker that predicts poor global outcome is not automatically a marker of reduced responsiveness to therapy. The former may reflect irreversible injury burden. The latter requires evidence of effect modification.

Composite immune indices deserve special scrutiny. Ratios such as the neutrophil-to-lymphocyte ratio (NLR) and systemic immune-inflammation index (SII) are attractive because they can be calculated from routine blood counts. Stroke studies support associations with mortality and functional outcome (46–48), and TBI studies provide related prognostic observations (66, 67). Their biological meaning remains broad because circulating cell populations reflect systemic rather than specifically central inflammatory mechanisms. These indices may be useful as low-cost covariates, but they are unlikely to function alone as definitive stratifiers of rehabilitation responsiveness.

Another recurring problem is overinterpretation of statistically significant associations. Biomarker studies frequently report independent associations after multivariable adjustment, yet the incremental predictive value over clinically accessible variables is often modest. A marker may achieve statistical significance while adding little meaningful discrimination to models built from age, severity, lesion characteristics, or baseline impairment. This is particularly relevant in rehabilitation, where decision-making must justify additional cost, complexity, or delays in treatment initiation. A biomarker that does not materially change the predicted benefit, timing, or type of rehabilitation is unlikely to be adopted in practice.

These limitations do not invalidate biomarker research. They define the conditions under which it must mature. Repeated sampling, phase-specific interpretation, transparent assay reporting, careful injury phenotyping, and explicit linkage to rehabilitation endpoints are all required if inflammatory biomarkers are to move from descriptive science to clinical stratification. The current literature suggests that this transition is feasible, but it also makes clear that no biomarker should be considered rehabilitation-ready unless it has been tested in cohorts where treatment exposure is well characterized and response is measured in a biologically meaningful way.

## 5 Rehabilitation modalities in biologically heterogeneous recovery

### 5.1 Exercise, robotics, and VR in biologically heterogeneous recovery

Among the rehabilitation modalities considered, therapeutic exercise has the broadest mechanistic and human literature relevant to inflammation. TBI exercise immunology provides an initial biological foundation (28), and translational stroke work supports effects on neuroplasticity and repair (30). A small single-center randomized trial in mild-to-moderate ischemic stroke measured serial inflammatory markers alongside very early exercise and subsequent functional outcomes (33). The study links intervention, biological change, and recovery more directly than prognostic cohorts, but it did not test whether baseline inflammatory status modified the comparative treatment effect or establish a selection threshold. Recent human synthesis further addresses neuroplasticity markers after stroke (35).

Current evidence does not establish that inflammatory status identifies responders to exercise-based rehabilitation after stroke or TBI. The randomized stroke study provides emerging direct interventional biomarker evidence (33), yet its small sample, very early treatment window, and absence of a prespecified biomarker-by-treatment interaction limit inference. Observational human and preclinical findings support the hypothesis that persistent inflammatory burden, systemic illness, fatigue, autonomic instability, or impaired trophic signaling may constrain dose tolerance or consolidation (35, 57). These variables should be tested against documented exercise dose and domain-specific gain; they should not currently determine whether exercise is prescribed.

Robotic systems can increase practice intensity and standardize movement training, but the clinical evidence base is derived predominantly from stroke cohorts. Contemporary syntheses address upper-limb efficacy and device context (69, 70). An umbrella review reported that statistically detectable gains did not reach established minimum clinically important differences and highlighted substantial heterogeneity (71). A network meta-analysis further examines comparative program effects (72), while additional upper-limb and gait-oriented syntheses address broader efficacy questions (73–75). None of these studies establishes inflammatory treatment-effect modification. Proposed links among inflammatory burden, practice tolerance, network reserve, and consolidation are conceptual inferences that require prespecified interaction testing, not criteria for current device selection.

VR can enhance engagement, task salience, access, and practice frequency. Stroke evidence includes VR-enabled telerehabilitation (76), broader remote-care syntheses (77–79), and additional service-level and umbrella evidence (80, 81). Recent VR-specific meta-analytic evidence addresses upper-limb outcomes (82). An umbrella review addresses the wider efficacy landscape (83). These findings concern average rehabilitation effects, not inflammatory stratification. Hypothesized associations with sensory-load tolerance, fatigue, vestibulo-autonomic symptoms, or cognitive effort remain mechanistic or conceptual. Trials should quantify actual exposure, adverse symptoms, retention, and clinical phenotype before testing whether serial inflammatory measures add predictive value. No current inflammatory profile can select VR intensity, immersion, or delivery format.

Group-average equivalence can coexist with clinically important heterogeneity, but existing studies do not show that inflammation explains that heterogeneity. Lower-intensity graded exercise, shorter practice bouts, or symptom stabilization may be clinically appropriate for selected patients on conventional grounds; attributing those needs to a measured inflammatory profile remains hypothetical. Future trials should distinguish standard clinical pacing from biomarker-defined treatment-effect modification.

This distinction does not diminish robotics or VR; it defines the next research question. Trials should test whether prespecified biological variables improve prediction of who benefits, at which phase, and at what dose beyond clinical and structural predictors. Table 2 separates established modality evidence from the current evidence level for inflammatory modification.

**Table 2:** Rehabilitation modalities, hypothesized inflammatory modifiers, candidate response patterns, practical research implications, and evidence level.

Modality	Plausible inflammatory modifiers	Candidate responder pattern	Candidate constrained-response pattern	Practical research implication	Evidence level for inflammatory modification
Therapeutic exercise: mechanistic foundation (28, 30); direct stroke trial (33); supporting syntheses (35, 57)	Cytokine tone, fatigue, autonomic regulation, vascular/trophic responsiveness	Stable symptoms, improving tolerance, capacity for repeated practice	Marked fatigue, post-exertional worsening, autonomic instability, unresolved systemic inflammation	Prospectively test serial biomarkers against dose tolerance and treatment gain	One small stroke trial provides direct interventional evidence for biomarker change alongside outcomes (33), but no prespecified biomarker-by-treatment interaction; other support is indirect or mechanistic
Robotics efficacy and program context (69, 70); umbrella and network syntheses (71, 72); additional stroke evidence (73–75)	Hypothesized effects on network efficiency, effort tolerance, and consolidation	Preserved corticospinal tract (CST) reserve, stable medical state, capacity for intensive repetition	Persistent dysregulation, low tolerance, fluctuating engagement despite nominal dose	Test inflammatory state as a prespecified effect modifier; do not use it for current device selection	Conceptual inference; robotics efficacy evidence is mainly stroke-derived and separate from inflammatory modification
Stroke virtual reality (76, 82, 83); traumatic brain injury (TBI) technology evidence (100–102)	Hypothesized links with sensory-load tolerance, fatigue, and vestibulo-autonomic symptoms	Stable symptoms, good engagement, retained adaptive capacity	Sensory overload, headache, dizziness, severe fatigue, unstable TBI symptoms	Measure symptom burden, exposure, and biological state in prospective trials	Conceptual inference; no validated inflammatory stratification studies
Stroke telerehabilitation (76, 77, 80, 81); traumatic brain injury and acquired brain injury evidence (103–106)	Hypothesized links with sleep, pain, systemic illness, autonomic symptoms, and self-management	Stable symptoms, adherence, support, need for distributed practice	High fluctuation, limited support, low tolerance for unsupervised dose	Test whether serial biology adds information beyond clinical readiness and adherence	Conceptual inference; modality evidence is stronger in stroke than TBI
Stroke non-invasive brain stimulation (84, 85, 87, 88); traumatic brain injury evidence (107–110)	Synaptic gain, glial-neuronal signaling, excitability, phase-specific plasticity	Preserved network reserve and reliable pairing with task practice	Diffuse white-matter injury, biological instability, poor consolidation	Embed biomarkers prospectively as mechanistic or effect-modification variables	Preclinical/mechanistic and indirect human evidence; direct inflammatory treatment matching is absent
Multimodal programs: clinical context (67, 68); stimulation-pairing evidence (86–88)	Hypothesized influence on tolerable sequence and dose	Improving stability and capacity to transition from stabilization to intensification	Instability requiring symptom control before high-demand training	Test sequence and dose within enrichment/adaptive designs	Conceptual framework; not a validated clinical algorithm

Modality efficacy evidence is distinguished from evidence that inflammation modifies treatment response. CST, corticospinal tract; NIBS, non-invasive brain stimulation; TBI, traumatic brain injury; VR, virtual reality.

### 5.2 Neuromodulation, telerehabilitation, and multimodal dosing in biological context

Non-invasive brain stimulation (NIBS) is designed to alter excitability or network dynamics in injured neural systems. Stroke evidence includes protocol-level syntheses for repetitive transcranial magnetic stimulation and transcranial direct current stimulation (84), low-risk-of-bias evaluation of stimulation paradigms (85), and upper-limb motor evidence (86). Recent network meta-analysis compares transcranial direct current stimulation paired with different rehabilitation interventions (87), while preclinical work supports inflammation-related mechanistic plausibility (88). Broader transcranial direct current stimulation evidence addresses function and safety (89, 90). Domain-specific syntheses then address upper- and lower-limb outcomes (91, 92), swallowing and aphasia (93, 94), cognition (95, 96), and recent dysphagia, language, and mood evidence (97–99). These bodies of evidence do not validate inflammatory thresholds for stimulation selection; efficacy evidence and biomarker-guided effect modification must remain separate claims.

Whether inflammatory status explains NIBS response is unknown. Mechanistic plausibility and indirect human evidence justify prospective testing, but there are no validated inflammatory thresholds or profiles for selecting a stimulation protocol. Proposed contrasts between biologically permissive and dysregulated states are hypotheses that may explain part of observed heterogeneity; they should be evaluated as pre-specified interaction terms while accounting for lesion features, network reserve, task pairing, and dose.

Technology-assisted rehabilitation after TBI has a smaller evidence base than after stroke. Systematic reviews describe potential gains when VR is embedded in structured programs (100, 101), and balance or mobility evidence is summarized separately (102). These studies do not establish inflammatory treatment-effect modification. Injury severity, vestibular symptoms, cognition, fatigue, headache, interface demand, and supervision may each explain outcome heterogeneity. The suggestion that inflammatory or autonomic state alters tolerance of sensory load or repetition is a hypothesis requiring TBI-specific prospective validation.

Telerehabilitation improves access and continuity for many stroke populations, including virtual-reality delivery (76) and broader remote rehabilitation models (77). Service-level and umbrella syntheses further support feasibility and average clinical effectiveness (80, 81). Evidence in TBI is smaller: a recent comprehensive rehabilitation review provides context (103), while telehealth, remote-technology, and severe acquired-brain-injury studies address selected populations (104–106). No prospective study shows that inflammatory biomarkers modify response to remote vs. in-person delivery. Clinical readiness, safety, goals, support, and measured adherence—not an inflammatory test—should guide current delivery decisions.

In TBI, non-invasive brain stimulation evidence is less mature than in stroke. A recent scoping review maps the rehabilitation field (107), and a 2025 meta-analysis of randomized trials evaluates repetitive transcranial magnetic stimulation across TBI outcomes (108). Contemporary cognitive evidence examines stimulation combined with cognitive training (109), while broader non-invasive systems and translingual stimulation studies provide additional context (110, 111). Samples, injury phases, targets, and outcomes remain heterogeneous, and inflammatory status is generally not integrated. Reported efficacy or safety cannot therefore be interpreted as evidence for inflammation-guided protocol selection.

Multimodal sequencing may be useful because dose and modality can be adapted over time, but inflammation-informed sequencing remains a conceptual proposal. Scenarios in which patients transition from stabilization to robotics, VR, or NIBS according to serial biomarkers should therefore be tested prospectively and should not be interpreted as protocolized care.

## 6 Toward a responder/non-responder framework

### 6.1 Multimodal signatures of responsiveness

A clinically meaningful responder/non-responder framework cannot be built on inflammatory biomarkers alone. Responsiveness emerges from tissue biology, network reserve, behavioral phenotype, and treatment exposure. In stroke, corticospinal and clinical prediction work established the principle of sequential biomarker use (112, 113). Magnetic resonance imaging and external validation studies refine this prognostic approach (114, 115), while severe-impairment cohorts and updated clinical reviews provide additional context (116, 117). These data support multimodal outcome prediction, not the untested assumption that inflammatory profiles select a rehabilitation treatment.

Inflammatory markers could be tested as additions to this framework: they may distinguish patients with similar structural reserve but different biological trajectories, and serial sampling may capture changes not visible on static imaging. These are research hypotheses, and their value would depend on improvement over accessible clinical predictors. For example, a trial could test whether declining interleukin 6 (IL-6) and high-sensitivity C-reactive protein (hs-CRP) add information to corticospinal reserve, medical stability, fatigue, and treatment tolerance when predicting gain from high-dose upper-limb practice. The analysis would need to separate prognosis from true treatment-effect modification, account for infection and vascular comorbidity, and validate calibration in an independent cohort. This scenario is not a clinical recommendation and requires prospective assessment of incremental value and biomarker-by-treatment interaction.

The Predict Recovery Potential 2 (PREP2) algorithm illustrates how clinical and neurophysiological measures can support a sequential, interpretable prediction model after stroke (112). Related biomarker, imaging, and motor-outcome studies define the surrounding prognostic evidence (113, 114), and recent external validation clarifies performance and the influence of cognitive syndromes (115). Severe-impairment imaging cohorts and updated biomarker reviews provide additional context (116, 117). These models predict recovery; they do not demonstrate inflammatory treatment matching. Any inflammation-informed extension must show incremental value over accessible clinical and structural predictors and must prospectively test whether the added information changes comparative treatment benefit.

Rehabilomics frameworks provide the broader rationale for combining biological and rehabilitation data after stroke and TBI (118, 119). Within that framework, wearable technologies can quantify gait, mobility, activity, asymmetry, and practice exposure with greater temporal density than clinic visits (120). Responsiveness unfolds over days and weeks, with fluctuations related to fatigue, sleep, systemic illness, dose, or biological instability. Serial inflammatory sampling paired with real-world performance could therefore test signatures of improvement, plateau, or vulnerability that sparse assessments miss. This remains a research use of digital outcomes rather than evidence that a fluctuation is inflammatory in origin.

Sources of heterogeneity that may separate response trajectories are summarized in Table 3. They include inflammatory state and structural reserve, but also age, frailty, vascular and endocrine comorbidity, extracranial injury, pain, cognition, attention, mood, sleep, and the distinction between restitution and compensation. A person who does not respond to one modality may be mismatched to its timing, dose, target, or delivery rather than globally non-responsive. Figure 3 presents a conceptual multimodal stratification framework that combines repeated biomarkers with imaging, clinical phenotype, treatment exposure, and digital outcomes. The higher, intermediate, and constrained response probabilities are illustrative rather than clinically calibrated estimates; no treatment-matching algorithm shown in the figure has been validated.

**Table 3:** Sources of heterogeneity separating responders from non-responders, with the evidence basis for each domain.

Domain of heterogeneity	Illustrative features	Why it can alter response	How to characterize it	Implication for trials and practice	Evidence basis
Inflammatory phase: stroke context (36, 37); biomarker timing (47, 48); traumatic brain injury (TBI) framework (53)	Hyperacute, acute, subacute, chronic persistence or resolution	A treatment may have different biological meaning at different phases	Repeated biomarkers plus timing and symptom trajectory	Use longitudinal re-stratification in research rather than fixed labels	Observational human plus mechanistic evidence
Condition and injury biology: rehabilitation (27); stroke markers (41, 42, 50); TBI prognosis (65)	Ischemic vs hemorrhagic stroke; mild vs moderate-to-severe TBI; extracranial trauma	Different mechanisms generate different inflammatory and recovery constraints	Subtype, imaging burden, mechanism, medical context	Do not pool biologically dissimilar populations without stratification	Direct clinical characterization plus observational biomarker evidence
Structural and network reserve: robotics and gait (73–75); prediction models (112, 115); validation (116, 117)	CST integrity, lesion topology, white-matter burden, network reserve	Reserve limits recruitment of restitution-capable circuits	Magnetic resonance imaging (MRI), neurophysiology, clinical motor testing	Combine reserve and inflammatory variables in prospective models	Human prognostic evidence; inflammatory integration remains conceptual
Systemic physiology and comorbidity (29, 41); stroke indices (46–48); TBI and neurobehavioral context (66, 99)	Frailty, obesity, diabetes, infection, sleep, pain, endocrine dysfunction	These factors affect biomarker interpretation and treatment tolerance	Medical review, laboratory data, symptom and medication tracking	Model confounding explicitly and preserve clinical interpretability	Observational human evidence
Neurobehavioral burden: TBI biomarker context (59, 65, 66); rehabilitation evidence (73, 103)	Cognition, mood, attention, fatigue, arousal, sensory overload	Behavioral capacity affects adherence, learning, and transfer	Clinical scales, neuropsychology, ecological observation	Match dose clinically; test biomarker-added value separately	Human clinical evidence; inflammatory linkage mostly indirect
Treatment exposure: biomarker context (58, 63, 67); modality evidence (83, 85, 88); digital dose (120)	Dose, intensity, timing, repetition, modality pairing	Average effects can hide exposure-response heterogeneity	Dosing logs, wearables, protocol-fidelity metrics	Prespecify dose and sequence in effect-modification analyses	Direct rehabilitation evidence for dose; biomarker interaction largely conceptual
Outcome architecture: stroke rehabilitation (76, 77, 85); TBI technology (100, 102, 106); digital context (120)	Restitution vs compensation, retention, daily-life transfer, home support	A patient may respond in one domain and not another	Modality-specific endpoints, digital outcomes, longer follow-up	Define response as multidimensional and time-dependent	Direct outcome-measurement evidence; inflammatory linkage indirect

Inflammatory integration is labeled as indirect or conceptual where direct treatment-effect modification has not been tested. CST, corticospinal tract; MRI, magnetic resonance imaging; TBI, traumatic brain injury.

**Figure 3:**
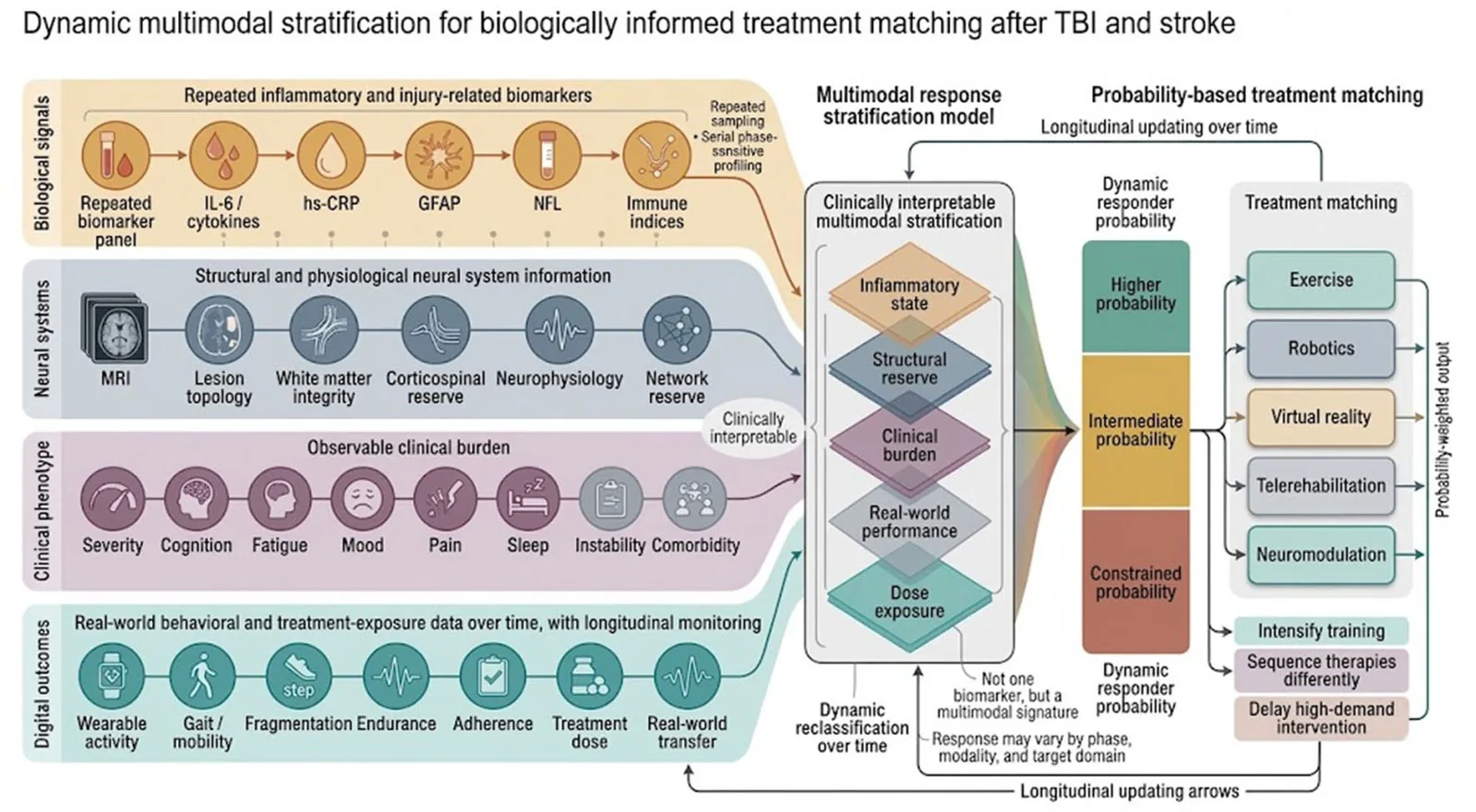
Multimodal responder/non-responder stratification framework integrating repeated biomarkers, neural-system measures, clinical phenotype, treatment exposure, and digital outcomes. Longitudinal updating is proposed to estimate treatment-specific response probabilities rather than assign fixed responder labels. The framework is intended for prospective validation and is not a clinically validated treatment-matching algorithm. GFAP, glial fibrillary acidic protein; hs-CRP, high-sensitivity C-reactive protein; IL-6, interleukin-6; MRI, magnetic resonance imaging; NfL, neurofilament light chain; TBI, traumatic brain injury.

Digital and wearable outcomes remain valuable because they can quantify instability, endurance, activity fragmentation, adherence, and transfer beyond the clinic (120). This measurement role has direct human relevance, whereas the proposed coupling of digital trajectories with inflammatory profiles remains untested. Serial biomarker sampling paired with treatment-exposure data could examine whether fatigue, post-exertional recovery, or day-to-day performance fluctuations carry biological information beyond symptoms and dose. Such studies should avoid treating a transient decline in home activity as proof of inflammatory constraint and should pre-specify which digital changes represent clinically meaningful response.

Another implication is that stratification must remain clinically interpretable. Complex multimodal models are attractive, but black-box prediction will not be enough for rehabilitation planning. Clinicians need to know whether a patient appears limited primarily by structural disconnection, inflammatory dysregulation, cognitive-behavioral burden, insufficient dose, or some combination of these factors. The most useful responder framework will therefore be one that improves decisions rather than merely improving statistical classification. It should tell the team whether to intensify training, simplify the behavioral target, sequence therapies differently, or delay a high-demand intervention until a more permissive window is reached. This matters because even dynamic clinical prediction models lose precision in some early and severely impaired stroke populations, suggesting that added biomarkers may be useful only if they improve interpretability as well as accuracy (121).

### 6.2 Designing enrichment-driven trials for precision neurorehabilitation

If rehabilitation responsiveness is biologically heterogeneous, then trial design must evolve accordingly. Conventional parallel-group trials that enroll broad stroke or TBI populations and apply a single intervention dose to all participants are vulnerable to dilution. True responders may be obscured by biologically mismatched patients, while negative average results can discourage development of interventions that may be highly effective in a defined subgroup. Enrichment strategies are therefore not a luxury. They are a methodological necessity for a field seeking precision rather than average-case care.

A future inflammation-informed enrichment strategy could pre-specify biological phase, randomization strata, repeated sampling, and treatment-by-biomarker interactions. Entry criteria might define a recovery window without assuming that a single threshold is universally favorable. Repeated measures could test whether biological change mediates or predicts treatment gain, while outcomes distinguish early improvement, retention, and real-world transfer. As a hypothetical design, not a treatment recommendation, a trial might stratify subacute stroke participants by corticospinal reserve and inflammatory burden, then compare alternative sequencing of symptom-limited stabilization and high-dose robotics. The design would also need a clinical-only comparator model to establish whether biomarker information is incremental.

Dose-response is equally important. Rehabilitation trials often emphasize intervention category while underestimating the importance of dose, intensity, and sequencing. Recent syntheses suggest that higher doses of task-oriented therapy may be required to maximize upper limb recovery after stroke, but dose effects are unlikely to be uniform across all patients (77, 118). Biological modifiers may help explain why some patients benefit from higher dose while others plateau, fatigue, or disengage. The implication is not that biological stratification replaces dosing science. It means dosing science should be biologically informed.

Adaptive designs may be especially valuable in this context because they allow preplanned modification based on accumulating information while preserving trial integrity (118). A trial might begin with broad enrollment but adaptively enrich toward biomarker-defined subgroups showing signal. Alternatively, dose or sequencing could be adjusted according to early biological and digital response patterns. This strategy is attractive for stroke and potentially for TBI because both conditions exhibit strong interindividual variability and often produce early clues about which pathways of recovery are available. Yet adaptive rehabilitation trials remain uncommon, and inflammatory biomarkers are rarely incorporated into their logic.

The translational path toward precision neurorehabilitation therefore requires a shift in what counts as a rehabilitation endpoint. Traditional scales remain essential, but they are not sufficient. Future trials should include biologically anchored outcomes such as repeated inflammatory measurements, neurophysiological markers of corticospinal function, imaging indicators of reserve, and digital measures of daily performance and adherence. Mechanistic measures should not be added as decorative secondary endpoints. They should be embedded to test whether the hypothesized biological pathway is actually altered by the intervention.

TBI-specific validation is especially important because injury mechanism, severity, extracranial trauma, imaging burden, cognition, and chronic sequelae vary substantially. A profile dominated by diffuse axonal injury and neurobehavioral instability poses different constraints from one dominated by persistent post-concussive symptoms, yet neither profile maps uniquely onto an inflammatory state. At present, proposed inflammatory profiles for selecting home-based VR, supervised pacing, or combined NIBS and task practice remain conceptual and should not be extrapolated from stroke. Stroke trials may offer design precedents for stratification, dosing, and outcome capture, but precision treatment claims require condition-specific prospective evidence. Multidimensional outcomes are essential because response in gait, cognition, symptoms, or participation may diverge within the same person.

Pragmatic implementation will also require repeated rather than one-time stratification. Recovery is dynamic. A patient classified as a likely non-responder in the early subacute phase may become a good candidate for intensive practice or stimulation later if inflammation stabilizes, fatigue improves, and network reserve is better expressed clinically. Precision neurorehabilitation should therefore avoid rigid labels. The aim is not to define permanent responders and non-responders, but to identify changing probabilities of benefit and to use those probabilities to sequence care more intelligently.

## 7 Discussion

### 7.1 What can already be said with confidence?

Several propositions can be stated, but with different levels of certainty. Neuroinflammation is part of post-injury recovery biology after stroke and TBI, as supported by condition-specific and cellular literature (9, 11). Recent work links chronic microglial states to neurorestoration in stroke (14), while TBI and cross-condition syntheses support phase-dependent biology (17, 23, 24). Evidence that inflammation modifies benefit from a defined rehabilitation treatment is far more limited. Prognostic association must not be conflated with treatment-effect modification. Inflammatory variables are therefore research candidates for multimodal stratification, not stand-alone selectors of modality, dose, or timing.

Is inflammation always detrimental to rehabilitation responsiveness? No. This is one of the most important misconceptions to avoid. Early and appropriately regulated inflammatory activity contributes to debris clearance, neurovascular signaling, and repair. The translational problem is not inflammation per se but the persistence, mistiming, or maladaptive configuration of inflammatory responses. A clinically useful framework must therefore distinguish inflammatory burden from inflammatory phase and must avoid equating “higher” with uniformly “worse” without context.

Can one blood test identify rehabilitation responders? At present, no. Stroke interleukin 6 evidence is predominantly prognostic (41), and high-sensitivity C-reactive protein reflects broad vascular or systemic burden (42, 43). Composite immune indices remain nonspecific in stroke (46–48). Glial fibrillary acidic protein and neurofilament light chain principally characterize glial or neuroaxonal injury (57–59, 64), while TBI interleukin 6 and immune-index studies remain prognostic (65–67). None has adequate prospective treatment-interaction evidence. Potential value lies in research panels interpreted with imaging, neurophysiology, clinical phenotype, and actual treatment exposure, not in isolated clinical thresholds.

Recent retrospective data from 198 critically ill patients with acute ischemic stroke associated higher SII and systemic inflammatory response index (SIRI) with poorer functional status at discharge, and higher SII with 28-day mortality (122). Because rehabilitation exposure and biomarker-by-treatment interaction were not assessed, this study strengthens short-term prognostic evidence rather than evidence for rehabilitation treatment matching.

Do current trials already support inflammation-based treatment matching? Only partially. Direct evidence is no longer wholly absent: one small single-center randomized stroke study linked very early exercise, serial inflammatory changes, and functional outcomes (33). However, it did not test a pre-specified baseline biomarker-by-treatment interaction, establish a treatment-selection threshold, or provide external validation. Most modality studies remain efficacy trials without inflammatory phenotyping, and most biomarker studies remain prognostic cohorts without standardized rehabilitation exposure. The evidence therefore supports prospective interaction testing, not current inflammation-based assignment.

The strength of this review is its integrative focus on rehabilitation responsiveness rather than general prognosis. Considering stroke and TBI together highlights shared biological questions, while explicit evidence labels and condition-specific conclusions prevent stroke-derived confidence from being extended to TBI without qualification. Recent translational studies were prioritized, with older studies retained only when foundational.

Its limitations should also be explicit. This is a narrative review and does not provide the exhaustive retrieval, formal bias assessment, or quantitative pooling expected of a systematic review. The available literature is uneven across conditions and modalities, with stroke far better represented than TBI in most rehabilitation domains. Biomarker studies are frequently limited by sampling heterogeneity, non-standardized assays, and outcomes that are not intervention-specific. Not all included studies are equally informative for responder stratification, and in several domains the argument remains stronger at the level of indirect human evidence or mechanistic plausibility than at the level of direct interventional testing. As a result, the proposed responder/non-responder framework is currently more advanced as a translational model than as a validated clinical algorithm.

The resulting framework should therefore be read as a structured research agenda: whether inflammation adds useful information to established clinical, structural, and treatment-exposure variables remains to be demonstrated.

### 7.2 What still blocks translation, and how should the field respond?

What still blocks translation is not a lack of plausible biology. It is the lack of harmonized methodology. Studies need standardized sampling windows that reflect clinically meaningful recovery phases rather than arbitrary convenience timepoints. They need transparent assay reporting, better separation of central from systemic sources of inflammation, and more careful characterization of ischemic vs. hemorrhagic stroke, mild vs. moderate-to-severe TBI, extracranial injury, infection, medication exposure, and comorbid inflammatory burden. Without that groundwork, inflammatory biomarker findings will continue to look promising yet remain difficult to compare or apply.

A second bottleneck is outcome selection. Rehabilitation studies often use broad disability scales that are necessary but insufficient. Future work should pair these with modality-specific endpoints, dose metrics, retention measures, and real-world activity capture. An intervention designed to enhance upper limb restitution should not be evaluated only by a generic global score. Likewise, a biomarker study that claims rehabilitation relevance should demonstrate how the marker relates to actual treatment exposure and gain, not just to spontaneous outcome.

A third bottleneck is trial architecture. Enrichment strategies, adaptive designs, repeated biomarker sampling, and multimodal endpoints are still uncommon in neurorehabilitation trials, despite being well suited to a heterogeneous biological process like recovery (118). The next generation of studies should be explicitly designed to test effect modification. They should ask whether patients with defined inflammatory and structural signatures respond differently to exercise, robotics, VR, telerehabilitation, or neuromodulation. They should also distinguish between early improvement, sustained benefit, and transfer into daily life.

Responsiveness should remain multidimensional and time-dependent rather than a permanent binary label. A patient may improve in gait endurance but not arm dexterity, or may tolerate a demanding modality only after medical and symptom stability changes. This complexity is clinically meaningful, but it also raises the evidentiary threshold for biomarker claims: an inflammatory measure must improve prediction for a pre-specified target, dose, and time window rather than correlate non-specifically with global outcome. The purpose of a responder framework is to test better decisions, not to relabel heterogeneous recovery.

Five practical priorities follow from the evidence gaps: harmonize phase-specific sampling and preanalytics; measure actual rehabilitation dose and modality-specific gain; prespecify biomarker-by-treatment interactions; integrate parsimonious biomarkers with structural, clinical, and digital measures; and externally validate models separately in stroke and TBI. These priorities require common data elements, clinically interpretable endpoints, and multicenter procedures that can distinguish a reproducible signal from site-specific assay or treatment effects. Response should be modeled as multidimensional and time-dependent rather than as a permanent binary label. Table 4 distinguishes literature-supported gaps from author-proposed research priorities, and Figure 4 presents these priorities as a staged roadmap rather than an implementation pathway.

**Table 4:** Evidence-based translational gaps and author-proposed future research priorities for precision neurorehabilitation.

Translational domain	Evidence-based finding (with references)	Evidence level	Author-proposed future research priority	Current clinical status
Sampling windows	Stroke timing (47, 48), traumatic brain injury (TBI) biomarker timing (51–53), and modality context (99, 102) show the limitations of phase mixing	Observational human and methodological synthesis	Adopt common phase definitions and serial sampling in prospective rehabilitation cohorts	Research only
Assays and pre-analytics	Neurovascular and biomarker methods (49, 53) and assay-domain evidence (56–58, 62) remain insufficiently harmonized	Methodological and observational evidence	Qualify assays and preanalytics for multicenter rehabilitation use	Research only
Panels vs. isolated markers	Inflammatory-marker evidence (43–45) and multimodal rationale (53, 68) show limited specificity for rehabilitation decisions	Observational/translational evidence	Compare parsimonious multimarker panels with clinical-only models	No validated panel for treatment assignment
Reserve and phenotype	Motor-reserve evidence (73, 112) and validation studies (115, 117) are often separated from multimodal and digital integration (118, 120, 121)	Human prognostic evidence; integration incompletely tested	Test incremental value and calibration of clinically interpretable multimodal models	Research only
Rehabilitation-specific endpoints	Many biomarker studies use mortality or broad disability rather than treatment exposure and gain (50, 85, 100, 120)	Observational and methodological evidence	Pair biomarkers with dose, rate of gain, retention, and real-world transfer	Research only
Enrichment/adaptive trials	Direct interaction tests remain rare across remote and stimulation evidence (81, 83, 85, 88) and TBI technology studies (101, 102)	Limited direct interventional evidence	Prespecify effect modifiers, serial sampling, and adaptive or stratified designs	Not ready for biomarker-led allocation
Digital monitoring	Remote stroke studies (76–79) and digital-measurement evidence (120, 121) show that sparse visits can miss exposure, fatigability, and transfer	Human measurement evidence	Link serial biological sampling to wearable and home-based outcomes	Research adjunct
Implementation discipline	Inflammatory treatment matching remains unvalidated in stroke stimulation evidence (91, 95, 99) and TBI neuromodulation studies (107, 108, 111)	Conceptual inference with indirect human/preclinical support	Require external validation, incremental utility, feasibility, and treatment-effect modification before implementation	Do not assign treatment from an isolated biomarker

Citations support the evidence-based findings and documented gaps; future research priorities are author-proposed and are not validated clinical guidance. TBI, traumatic brain injury.

**Figure 4:**
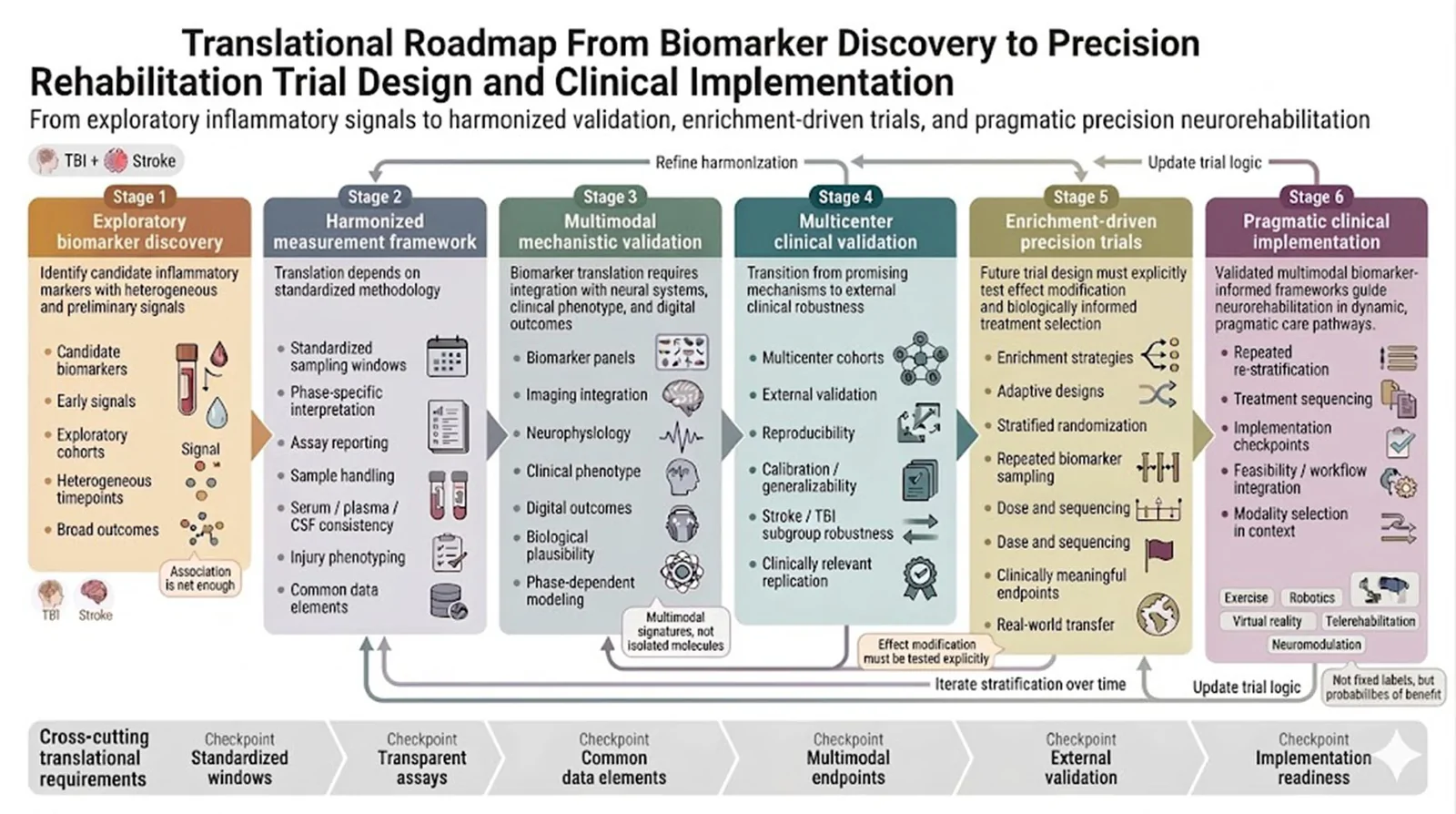
Translational roadmap from exploratory biomarker discovery to pragmatic precision-neurorehabilitation implementation. Six stages emphasize harmonized measurement, multimodal mechanistic validation, multicenter external validation, enrichment-driven trials, and implementation readiness. The sequence represents author-proposed research priorities and not an evidence-based clinical pathway. CSF, cerebrospinal fluid; TBI, traumatic brain injury.

Current evidence does not support assigning a device, exercise dose, remote-delivery format, or stimulation protocol from an isolated inflammatory value. Routine clinical decisions should continue to rely on established assessments of medical stability, symptoms, function, goals, support, and treatment tolerance. Biomarker-guided treatment matching should remain research-only until prospective studies demonstrate reproducible incremental utility and treatment-effect modification.

## 8 Conclusion

Available evidence supports neuroinflammation as a plausible, phase-dependent correlate of recovery biology after stroke and TBI; it does not yet establish inflammation as a modifier of benefit from any specific rehabilitation modality. No single biomarker or panel can currently classify rehabilitation responders, and inflammation-based treatment matching should remain confined to prospective research.

For stroke, human observational biomarker evidence and the rehabilitation evidence base are comparatively developed, particularly for exercise, robotics, VR, telerehabilitation, and NIBS. However, studies directly testing biomarker-by-treatment interactions remain scarce. Inflammatory measures may therefore be considered candidate enrichment variables for trials, not clinical selectors of modality, timing, or dose.

For TBI, evidence is substantially weaker and more heterogeneous across injury severity, phase, extracranial injury, and outcome domain. Current links between inflammatory profiles and modality-specific responsiveness are mainly indirect or mechanistic; extrapolation from stroke is not justified without TBI-specific prospective validation.

Progress in both conditions requires harmonized sampling, standardized assays, repeated measurements, rehabilitation-specific endpoints, and prespecified effect-modification analyses. Until reproducible incremental utility is demonstrated, the multimodal inflammation-informed framework should be regarded as a testable translational hypothesis rather than an implementation-ready algorithm.

## Data Availability

The original contributions presented in the study are included in the article/Supplementary material, further inquiries can be directed to the corresponding author.
