# Supplementary material for: Inflammatory profiling for precision neurorehabilitation after traumatic brain injury and stroke: a narrative review
Source: Front Neurol. 2026 Sep 24;17:1854973. doi: 10.3389/fneur.2026.1854973 (PMC13647574; doi:10.3389/fneur.2026.1854973)
Supplement: Supplementary file 1 [file Table_1.docx]

**Supplementary Material**

**PICO scope, search record, evidence charting, and Scale for the Assessment of Narrative Review Articles (SANRA) alignment**

**Supplementary Table S1. Population, intervention or exposure, comparator, and outcome (PICO) framework**

| **PICO element** | **Operational scope** | **Interpretive boundary** |
| --- | --- | --- |
| Population | Adults with ischemic or hemorrhagic stroke, or traumatic brain injury (TBI), across acute, subacute, and chronic recovery phases | Stroke and TBI are synthesized separately when evidence strength differs |
| Intervention or exposure | Inflammatory or injury-related profiling; therapeutic exercise; robotics; virtual reality (VR); telerehabilitation; non-invasive brain stimulation (NIBS); multimodal rehabilitation | Inflammatory state is treated as a candidate modifier, not an established treatment selector |
| Comparator | Alternative inflammatory state, usual care, alternative modality or dose, or no comparator when the source is descriptive or mechanistic | Absence of a comparator limits inference to association or biological plausibility |
| Outcomes | Treatment gain, dose tolerance, retention, real-world transfer, functional status, symptoms, cognition, participation, and adverse effects | Mortality or global prognosis is used only as indirect context unless rehabilitation exposure is measured |

**Supplementary Table S2. Information sources and search coverage**

| **Source** | **Coverage** | **Final search date** | **Purpose** |
| --- | --- | --- | --- |
| PubMed/MEDLINE | Database inception | March 23, 2026 | Biomedical and clinical studies; indexed reviews |
| Scopus | Database inception | March 23, 2026 | Multidisciplinary and citation coverage |
| Embase | Database inception | March 23, 2026 | Biomedical, pharmacological, and conference-indexed coverage |
| Web of Science Core Collection | Database inception | March 23, 2026 | Cross-disciplinary and citation coverage |
| Backward reference checking | Reference lists of high-relevance sources | Completed with final search update | Identification of foundational or directly relevant sources |

**Supplementary Table S3. Verbatim database search record.** The same three-block Boolean architecture was entered directly in PubMed, translated through the platform Query Builder for Embase and Web of Science, and executed in the Scopus Advanced Search interface.

| **Database/platform** | **Coverage** | **Final search date** | **Exact search string** |
| --- | --- | --- | --- |
| PubMed/MEDLINE | Inception to March 23, 2026 | March 23, 2026 | ("stroke" OR "ischemic stroke" OR "haemorrhagic stroke" OR "hemorrhagic stroke" OR "intracerebral hemorrhage" OR "traumatic brain injury" OR "brain trauma" OR "concussion") AND ("neuroinflammation" OR "inflammation" OR "inflammatory biomarker" OR "cytokine" OR "chemokine" OR "microglia" OR "astrocyte" OR "blood-brain barrier" OR "C-reactive protein" OR "interleukin-6" OR "glial fibrillary acidic protein" OR "neurofilament light chain" OR "neutrophil-to-lymphocyte ratio" OR "systemic immune-inflammation index" OR "systemic inflammatory response index") AND ("neurorehabilitation" OR "rehabilitation" OR "functional recovery" OR "neuroplasticity" OR "treatment response" OR "rehabilitation responsiveness" OR "responder" OR "exercise" OR "robot-assisted rehabilitation" OR "robotics" OR "virtual reality" OR "telerehabilitation" OR "non-invasive brain stimulation" OR "transcranial magnetic stimulation" OR "transcranial direct current stimulation" OR "neuromodulation" OR "wearable" OR "digital outcome") |
| Scopus (Advanced Search) | Inception to March 23, 2026 | March 23, 2026 | ( "stroke" OR "ischemic stroke" OR "haemorrhagic stroke" OR "hemorrhagic stroke" OR "intracerebral hemorrhage" OR "traumatic brain injury" OR "brain trauma" OR "concussion" ) AND ( "neuroinflammation" OR "inflammation" OR "inflammatory biomarker" OR "cytokine" OR "chemokine" OR "microglia" OR "astrocyte" OR "blood-brain barrier" OR "C-reactive protein" OR "interleukin-6" OR "glial fibrillary acidic protein" OR "neurofilament light chain" OR "neutrophil-to-lymphocyte ratio" OR "systemic immune-inflammation index" OR "systemic inflammatory response index" ) AND ( "neurorehabilitation" OR "rehabilitation" OR "functional recovery" OR "neuroplasticity" OR "treatment response" OR "rehabilitation responsiveness" OR "responder" OR "exercise" OR "robot-assisted rehabilitation" OR "robotics" OR "virtual reality" OR "telerehabilitation" OR "non-invasive brain stimulation" OR "transcranial magnetic stimulation" OR "transcranial direct current stimulation" OR "neuromodulation" OR "wearable" OR "digital outcome" ) |
| Embase (Query Builder) | Inception to March 23, 2026 | March 23, 2026 | ('stroke' OR 'ischemic stroke' OR 'haemorrhagic stroke' OR 'hemorrhagic stroke' OR 'intracerebral hemorrhage' OR 'traumatic brain injury' OR 'brain trauma' OR 'concussion') AND ('neuroinflammation' OR 'inflammation' OR 'inflammatory biomarker' OR 'cytokine' OR 'chemokine' OR 'microglia' OR 'astrocyte' OR 'blood-brain barrier' OR 'c-reactive protein' OR 'interleukin-6' OR 'glial fibrillary acidic protein' OR 'neurofilament light chain' OR 'neutrophil-to-lymphocyte ratio' OR 'systemic immune-inflammation index' OR 'systemic inflammatory response index') AND ('neurorehabilitation' OR 'rehabilitation' OR 'functional recovery' OR 'neuroplasticity' OR 'treatment response' OR 'rehabilitation responsiveness' OR 'responder' OR 'exercise' OR 'robot-assisted rehabilitation' OR 'robotics' OR 'virtual reality' OR 'telerehabilitation' OR 'non-invasive brain stimulation' OR 'transcranial magnetic stimulation' OR 'transcranial direct current stimulation' OR 'neuromodulation' OR 'wearable' OR 'digital outcome') |
| Web of Science Core Collection (Query Builder) | Inception to March 23, 2026 | March 23, 2026 | ((ALL=(("stroke" OR "ischemic stroke" OR "haemorrhagic stroke" OR "hemorrhagic stroke" OR "intracerebral hemorrhage" OR "traumatic brain injury" OR "brain trauma" OR "concussion") )) AND ALL=(("neuroinflammation" OR "inflammation" OR "inflammatory biomarker" OR "cytokine" OR "chemokine" OR "microglia" OR "astrocyte" OR "blood-brain barrier" OR "C-reactive protein" OR "interleukin-6" OR "glial fibrillary acidic protein" OR "neurofilament light chain" OR "neutrophil-to-lymphocyte ratio" OR "systemic immune-inflammation index" OR "systemic inflammatory response index") )) AND ALL=(("neurorehabilitation" OR "rehabilitation" OR "functional recovery" OR "neuroplasticity" OR "treatment response" OR "rehabilitation responsiveness" OR "responder" OR "exercise" OR "robot-assisted rehabilitation" OR "robotics" OR "virtual reality" OR "telerehabilitation" OR "non-invasive brain stimulation" OR "transcranial magnetic stimulation" OR "transcranial direct current stimulation" OR "neuromodulation" OR "wearable" OR "digital outcome")) |

**Supplementary Table S4. Eligibility, prioritization, and exclusion logic**

| **Domain** | **Included or prioritized** | **Excluded or de-emphasized** | **Rationale** |
| --- | --- | --- | --- |
| Conditions | Stroke and TBI with condition-specific interpretation | Other neurological conditions unless needed for a tightly bounded mechanistic point | Preserves etiological relevance |
| Evidence types | Human interventional and observational studies; systematic or scoping reviews; meta-analyses; consensus and methodological reports; selected mechanistic studies | Preclinical work without a direct link to inflammatory control of recovery, plasticity, or treatment response | Maintains translational focus |
| Rehabilitation relevance | Defined rehabilitation exposure, response, tolerance, dose, retention, transfer, or a biomarker domain necessary to interpret those constructs | Biomarker studies without relevance to recovery biology or rehabilitation-oriented phenotyping | Separates general biomarker cataloguing from the review question |
| Acute and prognostic studies | Retained when needed to define phase, measurement validity, biological context, or prognostic limits | Used as direct evidence of treatment selection when rehabilitation exposure was not measured | Prevents prognosis from being conflated with treatment-effect modification |
| Recency | Recent and directly relevant evidence, especially 2021–2026 | Older work superseded by current synthesis | Updates the evidence base while retaining foundational studies |
| Selection process | Purposive, iterative narrative selection and backward reference checking | Claims of exhaustive PRISMA-style screening or reconstructed counts | Matches the declared narrative-review design |

**Supplementary Table S5. Data charting and evidence-level classification**

| **Component or evidence label** | **Operational definition** | **Permitted interpretation** | **Principal limitation** |
| --- | --- | --- | --- |
| Data charting | Condition/subtype; phase; design; sample context; biomarker domain; modality and documented dose; outcome; prognostic versus response question | Supports structured comparison across heterogeneous sources | Not equivalent to systematic extraction or formal risk-of-bias assessment |
| Direct interventional evidence | Prospective human rehabilitation study relating biomarker state or change to a defined intervention and outcome | May link treatment, biology, and outcome within the tested protocol | Without a prespecified interaction, it does not establish biomarker-guided treatment selection |
| Observational human evidence | Biomarker association with severity, symptoms, function, prognosis, or tolerance | Supports association and biological phenotyping | Confounding and absence of randomized comparative treatment effects |
| Preclinical or mechanistic evidence | Experimental evidence connecting inflammatory pathways with plasticity, repair, excitability, or learning | Supports biological plausibility and target selection | Cannot establish clinical response or treatment thresholds |
| Conceptual inference | Author-generated link among inflammatory state, modality, dose, or responder trajectory not directly tested | Defines a testable hypothesis or trial-design priority | Must not be interpreted as current clinical guidance |

**Supplementary Table S6. SANRA-aligned quality map**

| **SANRA domain** | **How addressed** | **Manuscript location** | **Boundary** |
| --- | --- | --- | --- |
| Justification of importance | Clinical heterogeneity and the distinction between prognosis and treatment response are defined | Introduction | Does not imply treatment-effect modification is established |
| Statement of aims | Timing, biomarkers, modality-specific responsiveness, and trial translation are prespecified | Final Introduction paragraph | Broad integrative aim is appropriate to a narrative review |
| Description of literature search | Sources, date limits, concept architecture, reference checking, and verbatim-search fields are reported | Methods; Supplementary Tables S2 and S3 | No retrospective screened-record count |
| Referencing | Recent direct sources are prioritized; foundational studies are retained selectively; citations are assigned to specific claims | Throughout; References | No citation is treated as evidence beyond its design |
| Scientific reasoning | Direct interventional, observational human, mechanistic, and conceptual evidence are separated | Methods; modality sections; Supplementary Table S5 | Prognosis is not equated with treatment selection |
| Presentation of relevant data | Tables separate current findings, evidence level, future priorities, and clinical status | Tables 1–4 | No numerical SANRA self-score or formal risk-of-bias claim |
| Framework reference | Baethge C, Goldbeck-Wood S, Mertens S. SANRA—a scale for the quality assessment of narrative review articles. Res Integr Peer Rev. 2019;4:5. doi: 10.1186/s41073-019-0064-8. | Supplementary Material | SANRA is a critical-appraisal instrument, not a reporting guideline |
